# Determination of ^238^U and ^40^K Radionuclide Concentrations in Some Granite Rocks by Gamma Spectroscopy and Energy Dispersive X-ray Analysis

**DOI:** 10.3390/ma15155130

**Published:** 2022-07-23

**Authors:** Hanan Al-Ghamdi, M. A. El-Nahal, I. H. Saleh, Mohamed Elsafi, M. I. Sayyed, Aljawhara H. Almuqrin

**Affiliations:** 1 Department of Physics, College of Science, Princess Nourah bint Abdulrahman University, P.O. Box 84428, Riyadh 11671, Saudi Arabia; hmalghmdi@pnu.edu.sa (H.A.-G.); ahalmoqren@pnu.edu.sa (A.H.A.); 2 Department of Environmental Studies, Institute of Graduate Studies and Research, Alexandria University, Alexandria 21526, Egypt; igsr.nahalmoh@alexu.edu.eg (M.A.E.-N.); igsr.ihindawy@alexu.edu.eg (I.H.S.); 3 Physics Department, Faculty of Science, Alexandria University, Alexandria 21511, Egypt; mohamedelsafi68@gmail.com; 4 Department of Physics, Faculty of Science, Isra University, Amman 11622, Jordan

**Keywords:** radionuclide concentration, granite rocks, gamma-ray spectroscopy, EDX, ^238^U, ^40^K

## Abstract

Uranium-238 (^238^U) and potassium-40 (^40^K) are important naturally occurring radionuclides. Gamma spectroscopy is a direct, non-destructive method used to determine radionuclide concentrations, but it suffers from the interference of gamma lines. ^40^K gamma spectroscopy is affected by background interference, which leads to a reduction in the minimum detectable activity. The energy dispersive X-ray analytical technique is quick, with fewer interference problems or background effects. However, it is an indirect method for calculating and deducing the concentrations of isotopes. The aim of the present study was to compare and evaluate both techniques so that they can be utilized efficiently. The results of ^238^U and ^40^K were measured by well-calibrated gamma spectroscopy and energy dispersive X-ray techniques. the results indicated that Halayeb White granite is the most environmentally safe compared to the other two types because it contains a very low concentration of uranium 238 and potassium 40.

## 1. Introduction

Radiation exposure from natural and artificial sources is a persistent and unavoidable hazard. The major effects on humankind come from natural radiation sources, and the global average effective dose per person is 2.4 mSv per year. Natural sources comprise 80% of the total dose that humans receive [1]. The primary contribution is due to naturally occurring radioisotopes in the Earth’s crust, such as ^232^Th, ^238^U, and ^40^K. These radioisotopes exist extensively in the lithosphere and are found in mineral ores, soils, rocks, etc. Due to the break-down and weathering of rocks, ^40^K can be transferred to food pathways.

The average worldwide specific activity values of ^40^K, ^238^U, and ^232^Th in soil are 400, 37, and 33 Bq/kg, respectively. Cancer is one of the most detrimental radiation adverse health effects. Hence, the accurate determination of naturally occurring isotopes is essential for determining the radiation health hazard indices to estimate the risk level due to potential exposure to background radiation [2,3].

Previous studies have demonstrated various detection and measuring techniques to determine the concentration of ^40^K and ^238^U in different environmental matrices [4,5,6]. Gamma spectroscopy is the standard method used to measure the specific concentration of ^40^K in soil or natural rock due to the ^40^K gamma line at 1460.8 keV, with a considerable intensity of 10.66% [7]. On the other hand, alpha spectroscopy is a suitable method for directly determining specific ^238^U concentrations, but it suffers from complex chemical preparation and tracing methods prior to measuring. Hence, gamma spectroscopy dominates the detection process of ^40^K and ^238^U if the secular equilibrium between ^226^Ra (the last nongaseous daughter of ^238^U) is expected, as in the case of natural minerals and rocks such as granite [8].

Gamma spectroscopy is a simple and direct method that does not require chemical preparation and tracing complexities. However, it has the disadvantage of gamma line interference, as in the case of ^235^U and ^226^Ra at 185.7 and 186.2 keV, respectively. Furthermore, to obtain accurate results, gamma spectroscopy takes a long time to achieve sufficient counts to reduce uncertainty and statistical error, especially in the case of very low-concentration environmental tracing radioisotopes [9,10,11].

Using an energy dispersive X-ray (EDX) spectrometer to determine the elemental composition is very common during the analysis of environmental and geological samples. Deducing the radioisotope weight ratios from the elemental composition is theoretically possible by assuming that isotopic natural abundance ratios are preserved. The EDX technique is rapid, reliable, and does not require complicated sample preparation [12,13].

In this study, the gamma spectroscopy and EDX methods are discussed and compared in terms of detection limits, uncertainty, and accuracy. We illustrate the limitations of each analytical method and their most efficient uses to determine ^40^K and ^238^U concentrations in natural rocks, minerals, and environmental samples under the secular equilibrium of ^238^U and ^226^Ra by investigating three different types of granites rocks with expected various concentrations of ^40^K and ^238^U. The expected achievement of this comparative study is to determine when the technique is most efficient and what its limitations are.

## 2. Materials and Methods

### 2.1. Samples

**Figure 1:**
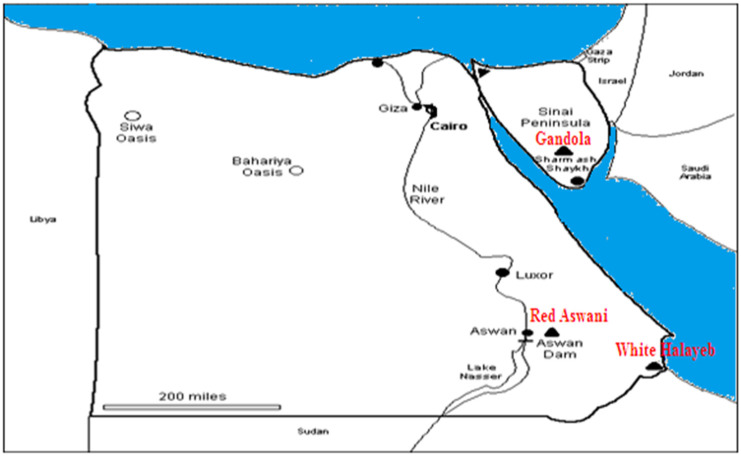
Map of the sample locations.

Three different natural granite types are shown in Table 1: Gandola, Red Aswani, and White Halayeb. The samples were collected from various places in Egypt, as shown in Figure 1. Five samples were obtained from each kind of granite rock. Small pieces of each type of granite were ground, and then the produced powder was stirred and mixed thoroughly until achieving a homogenous fine powder.

### 2.2. Gamma Spectrometer Method

**Figure 2:**
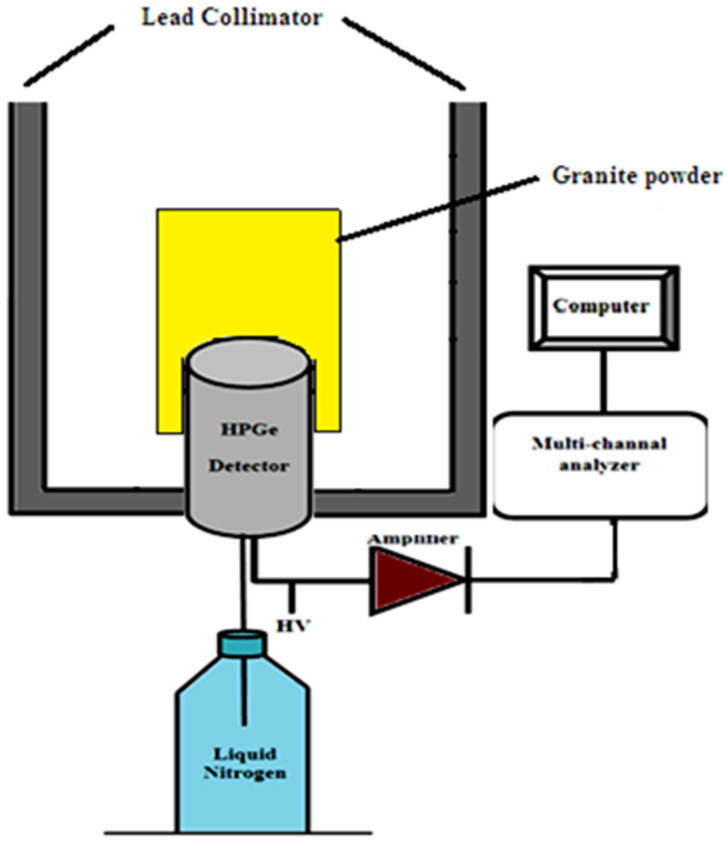
Illustration of the gamma spectrometer technique.

The radiation measurements were performed by using a high-resolution gamma spectroscopy system. It consisted of a high-purity germanium detector, the “HPGe” (see Figure 2) model CS20-A31CL, with a multi-channel analyzer (MCA) of 4022 channels at the Institute of Graduate Studies and Research, Alexandria University, Egypt. The detection system’s relative efficiency is 24.5% for 1333 keV of ^60^Co-line for the efficiency of the (3″ × 3″) NaI scintillation detector at 25 cm from the radiation source. The achieved energy resolution was 0.93 keV at the 122 keV gamma line and 1.95 keV at the 1333 keV gamma line of ^60^Co.

The secular equilibrium between ^238^U and ^226^Ra was utilized to determine the ^238^U concentration by using the 186.2 keV gamma line of ^226^Ra after correction to remove ^235^U interference. The specific activity (Bq Kg^−1^) for dry mass (*M*) was calculated by [14]:

(1) $$A_{S}=\frac{N_{T}}{\varepsilon \left(E\right)\times I_{\gamma }\left(E\right)\times M}$$

where *N_T_*, ε, and $I_{\gamma }\;$ are the total count rate, efficiency of the HPGe detector, and relative intensity of peak energy *E*, respectively. The total count rate of the 186.2 keV line includes ^226^Ra (186.2 keV, 3.555%) and ^235^U (185.7 keV, 57%), where $N_{T}=N_{226_{Ra}}+N_{235_{U}}$ and the relation between the total count rate and count rate of ^226^Ra is given by [15]:

(2) $$N_{226_{Ra}}=0.572\times N_{T}$$

Thus, we can calculate the specific activity of ^238^U using Equation (3) [16,17]:

(3) $${\left(A_{s}\right)}_{238_{U}}=\frac{0.572\times N_{T}}{\varepsilon_{238_{U}}^{186.2}\times {\left(I_{\gamma }\right)}_{238_{U}}^{186.2}\times M}$$

The minimum detectable activity (MDA) is related to detector sensitivity and can be defined as the smallest amount of activity distinguishable from the background, which can be quantified at a given confidence level (usually 95%). The minimum detectable activity was automatically calculated using the Genie 2000 data acquisition and analysis software [14], as follows:

(4) $$\mathrm{MDA}=\frac{1.65\times \sqrt{B}}{\varepsilon \times B.R\times T\times M}$$

**Figure 3:**
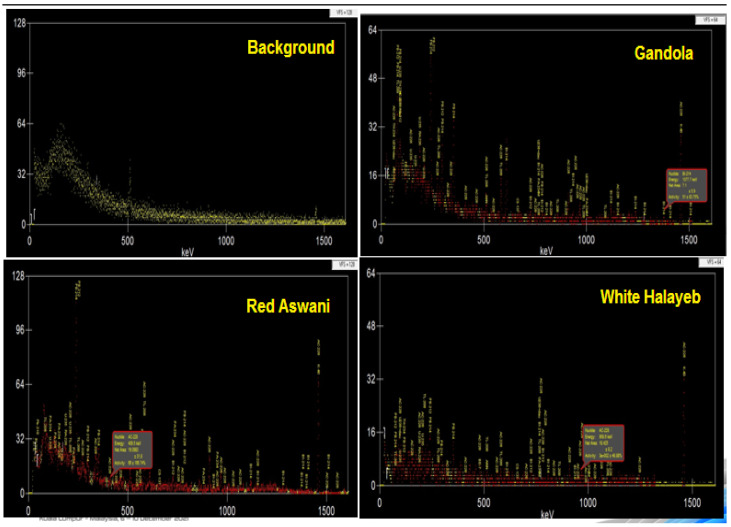
Experimental spectrums using the HPGe detector for background radiation and the three discussed granite samples.

Proper detector energy and efficiency calibrations were conducted before the measurement. The acquisition time was chosen to obtain sufficient counts under each photopeak so that the statistical uncertainty was below 1% (with a measuring time of 12 h). The spectrum was analyzed by using the Genie 2000 data acquisition and analysis software (made by Canberra), which is an integrated set for counting and analyzing spectra to calculate the net count rate at 186.2 keV and 1460 keV, as shown in Figure 3.

A 0.5 kg powder sample was enclosed in a 0.5 L Marinelli beaker to acquire the 4Π counting geometry. It was placed inside lead shielding to reduce the background and obtain a lower minimum detectable activity. The background was determined by allowing the detector to count a blank sample of an empty 0.5 L Marinelli beaker for long enough to acquire a good statistical result of the background measurement. Then, the background spectra were subtracted from the gross sample spectrum under each photo-peak of interest to obtain the net count of each photo-peak [18,19].

### 2.3. EDX Method

**Figure 4:**
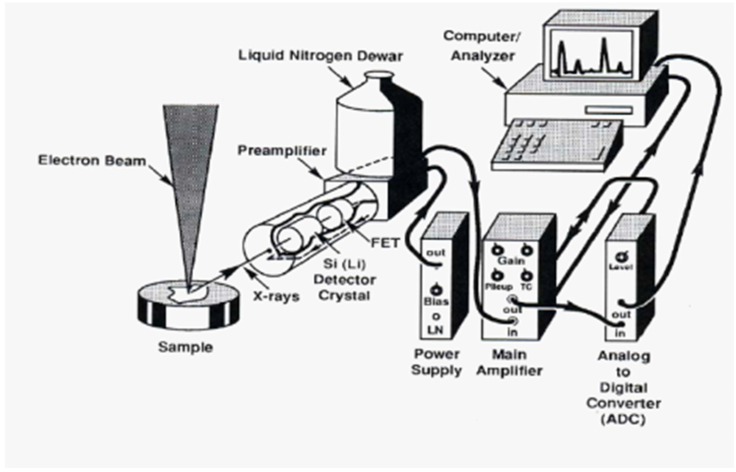
Schematic diagram of an EDX system.

The elemental potassium and uranium concentrations of the investigated samples were determined through the energy dispersion X-ray (EDX) unit of the electron scanning microscope (SEM) at the city of scientific research in Alexandria, Egypt. Uranium is a trace element in granites, making accurate detection with EDX difficult. Therefore, each sample was scanned by the electron microscope for multiple scans (with repeated measurements), targeting different regions of one sample to perform a comprehensive scan for each sample. The measuring time was selected to be the maximum time allowed by the instrument (30 min), while increasing the beam current as much as possible to ensure the accurate determination of the elemental concentration inside the sample. A schematic diagram of an EDX micro-analysis method is shown in Figure 4 [20].

The detection limit of an EDX system is affected by bremsstrahlung radiation—with lower concentrations, statistical errors and uncertainties are higher. Further, the detection limits for heavy elements (using the L or M lines) such as uranium tend to increase because the peak-to-background ratio is lower than it is for K lines [21]. The detection limit of the EDX system can be estimated from the following equation [22]:

(5) $$C_{\mathrm{DL}}\;\geq \frac{3\sqrt{N\left(B\right)}}{N\left(S\right)-N\left(B\right)}\;C_{s}$$

where *N(B)* is the average background count, *N(S)* is the average count of the standard, and *C_s_* is the concentration of the standard. The specific activity concentration of the radionuclides can be estimated theoretically from the elemental concentration given by EDX [23,24]:

As = λ × N (6)

where λ is the decay constant of the radionuclide and *N* is the number of radionuclides in a 1 kg granite sample, which can be given by [25]:

(7) $$N=\frac{C\times R\times N_{A}\times 1000}{W}\;$$

where *C* is the elemental concentration of mass percentage in the granite sample estimated from EDX, *R* is the isotopic abundance, *N_A_* is the Avogadro’s number, and *W* is the atomic weight of the radionuclide.

## 3. Results and Discussion

**Figure 5:**
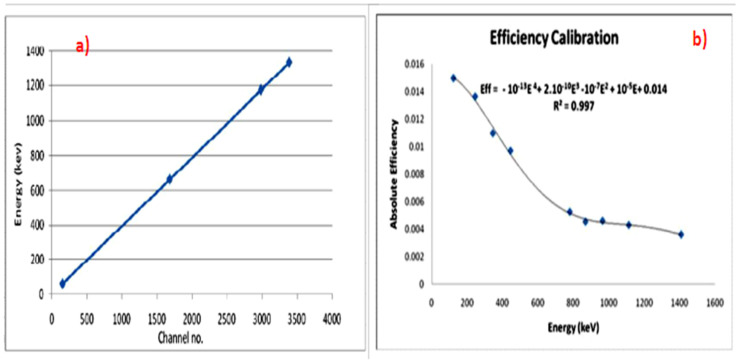
Calibration of HPGe detector. (**a**) Energy calibration. (**b**) Efficiency calibration.

First, the HPGe detector was calibrated (energy and efficiency calibration) using three point sources for the energy calibration—^241^Am (59.54 keV), ^137^Cs (661.65 keV), and ^60^Co (1173 and 1333 keV)—while the ^152^Eu volumetric source (121.8, 244.7, 344.3, 444, 778.9, 867.4, 964.0, 1112.1, and 1408.0 keV) for the efficiency calibration was as shown in Figure 5. From the efficiency calibration curve, as shown in Figure 5b, the equation for the efficiency as a function of energy was obtained. From this equation, the detector’s efficiency was calculated at 186.2 keV (^226^Ra) and 1460 keV (^40^K).

**Figure 6:**
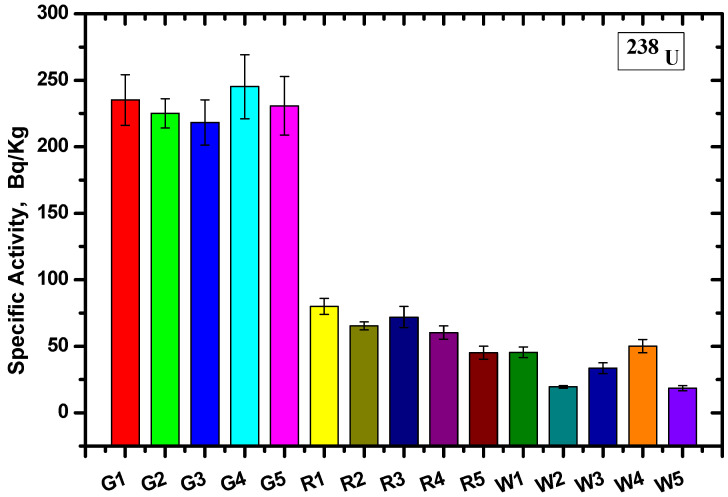
The specific activity for ^238^U using the Ra-226 γ-line at 186.2 keV for the different granite samples.

**Figure 7:**
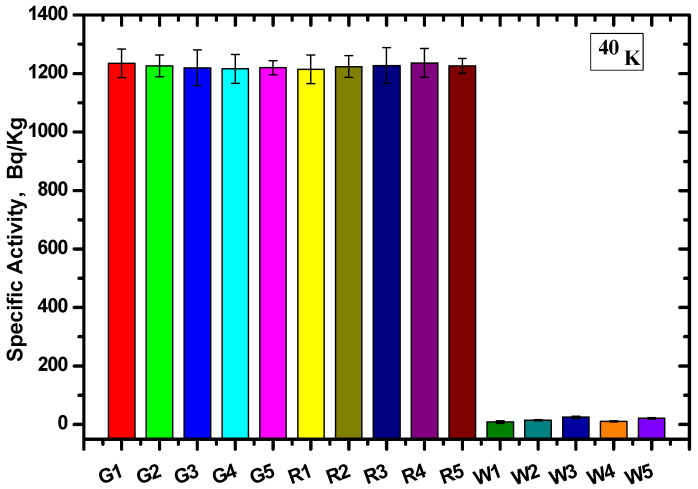
The specific activity for ^40^K at 1406 keV for the different granite samples.

Calculating the count rate and corresponding efficiency of each energy and substituting this into Equation (1), the specific activity of the different radionuclide (AS) samples and the average values were calculated. The values were determined for each site based on the gamma ray spectrometer method for U-238 and K-40 (Figure 6 and Figure 7, respectively).

The specific activity was calculated for five samples of each type, and the specific activity of U-238 for all Gandola samples was higher than the Red Aswani granite, while the lowest specific activity resulted from the white Halayeb granite. On the other hand, the specific activity was nearly equal for the K-40 for the Gandola and Red Aswani samples, and the activity was almost non-existent in the white Halayeb granite.

The results are tabulated in Table 2, including the average value for each granite type and the standard deviation. The results showed that the Gandola granite had the highest ^238^U concentration compared with the other two types of granite, while White Halayeb had the lowest concentration. Furthermore, the ^40^K concentration was nearly constant in both Gandola and Red Aswani, while the ^40^K concentration for White Halayeb was insignificant compared to Gandola and Red Aswani. The standard deviations (SDs) in the U-238 radionuclide were 10.2, 13.1, and 14.5 for Gandola, Red Aswani, and White Halayeb, respectively. Meanwhile, in K-40, the SDs were 8, 7.9, and 6.9 for Gandola, Red Aswani, and White Halayeb, respectively.

The minimum detectable activities of the radionuclides under study are listed in Table 3. The minimum detectable activity (MDA) of ^40^K was lower than the MDA of ^226^Ra. This might be due to the intensity of the ^40^K gamma line at 1460 (10.6%), which is higher than ^226^Ra at 186.2 keV (3.64%).

The absolute efficiency calibration of the gamma spectroscopy was validated using radioactive mixed standard sets in a soil matrix in 1000 mL Marinelli beakers. The quality control soil samples (supplied through the proficiency testing Mixed Analytic Performance Evaluator Program (MAPEP), organized by the Department of Energy in the United States) were measured in parallel with the analyzed samples to keep a bias of <5%, as in the laboratory criteria. Table 4 lists the most recent participation in the MAPEP program, which coincided with the sample measuring. Meanwhile, the EDX measurement accuracy was evaluated using the IAEA-312 soil matrix reference material for U determination and a high-analytical-purity potassium chloride for potassium measurement. The bias of the laboratory value was 3.6% for uranium and 1.5% for potassium.

**Figure 8:**
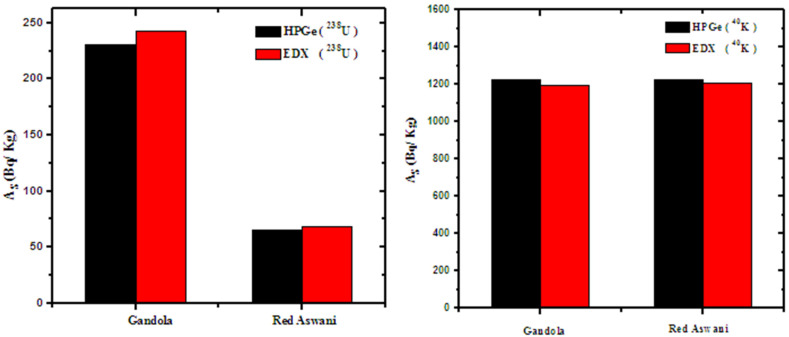
The specific activity by the two methods for Gandola and Red Aswani.

According to Section 2.3, the specific activity was calculated for the same samples and compared with the gamma spectroscopic results, as shown in Figure 8. The relative deviation was calculated between the two results and tabulated in Table 5. The relative deviation between the EDX analysis and gamma ray spectroscopy was determined as:

(8) $$R.D\left(\%\right)=\frac{{\left(A_{S}\right)}_{EDX}-{\left(A_{S}\right)}_{Gamma}}{{\left(A_{S}\right)}_{EDX}}\times 100$$

The results in Table 5 showed good agreement between the two methods, where the *R.D* in all types of rocks was ≤ 6%. This indicates the correctness of using the EDX analysis method without exposure to gamma rays when calibrating the device. The results indicated that EDX is an effective and reliable method for high concentrations, while gamma ray spectroscopy is more effective for low radionuclide contents, but with a high degree of uncertainty.

## 4. Conclusions

The concentrations of uranium-238 (^238^U) and potassium-40 (^40^K) in the three different types of granite (Gandola, Aswani Red, and White Halayeb) were determined for each type of the five samples using gamma ray spectroscopy and EDX analysis, and the average value was calculated. The results of the two methods showed a good agreement between the radionuclide concentration measurements. The results show that the EDX analysis method is fast, and it is recommended in the case of high concentrations, while gamma spectroscopy is more suitable in the case of low radionuclide content, but with a high degree of uncertainty. Finally, the results indicated that Halayeb White granite is the most environmentally safe compared to the other two types because it contains a very low concentration of uranium 238 and potassium 40.

## Figures and Tables

**Table 1:** Sample markings, descriptions, and locations.

Marking Code	Type of the Samples	Commercial Name	Quarry Sites
G1-G5	granite	Gandola	Sinai
R1-R5	granite	Red Aswani	Halayeb
W1-W5	granite	White Halayeb	Aswan

**Table 2:** Radionuclide specific activity (Bq/Kg) for the different samples by the gamma ray spectrometer method.

Granite Type	Sample	Radionuclide Specific Activity Bq/Kg	
		^226^Ra at 186.2 keV	^40^K at 1460 keV
Gandola	G1	235.2 ± 19	1235.5 ± 49
	G2	225.2 ± 11	1226.3 ± 37
	G3	218.3 ± 24	1219.7 ± 61
	G4	245.2 ± 22	1216.3 ± 49
	G5	230.7 ± 27	1220.2 ± 24
	SD	10.2	8.0
	Average	230.92 ± 21	1223.6 ± 44
Red Aswani	R1	80 ± 6	1214.8 ± 49
	R2	65.3 ± 3	1223.9 ± 22
	R3	72 ± 8	1227.7 ± 52
	R4	60.4 ± 5	1236.7 ± 41
	R5	45.2 ± 5	1226.5 ± 25
	SD	13.1	7.9
	Average	65 ± 6	1226 ± 44
White Halayeb	W1	45.5 ± 4	8.2 ± 1.5
	W2	19.5 ± 1	15.0 ± 1.7
	W3	33.6 ± 4	25 ± 3.5
	W4	50.2 ± 5	11 ± 1.6
	W5	18.5 ± 2	21 ± 2.3
	SD	14.5	6.9
	Average	33 ± 3	16 ± 2

**Table 3:** The minimum detectable activity of the radionuclides of interest.

Radionuclide	Ra-226	K-40
Energy (keV)	186.2	1460
MDA (Bq/Kg)	9.5	3.5

**Table 4:** The most recent participation in the MAPEP program.

Analyst	Result	Ref. Value	Bias %	Acceptance Range
Cesium-134	24.98	23.5	1.37	16.5–30.6
Cesium-137	21.09	19.1	1.53	13.4–24.8
Cobalt-57	33.31	29.9	2.5	20.9–38.9
Potassium-40	0.09		0.09	False-positive test
Zinc-65	15.53	18.3	1.61	12.8–23.8

**Table 5:** The elemental contents and equivalent specific activity for the three types of granites, as well as the relative deviation between the two presented methods.

Granite Rock Type	U Content (PPM)	Equivalent ^238^U Specific Activity (Bq/Kg)	R.D (%)
Gandola	20	243 ± 12	5.4
Red Aswani	7.25	68.5 ± 8	3.7
White Halayeb	Below detection limit	-	-
Granite Rock Type	K content (PPM)	Equivalent ^40^K specific activity(Bq/Kg)	R.D%
Gandola	37,700	1196.6 ± 3	2.2
Red Aswani	38,000	1206.2 ± 2	1.6
White Halayeb	Below detection limit	-	-

## Data Availability

Not applicable.
